# Diagnostic performance of the triglyceride-glucose index in predicting occurrence of cancer: a meta-analysis

**DOI:** 10.3389/fonc.2025.1532253

**Published:** 2025-05-09

**Authors:** I-Wen Chen, Wei-Ting Wang, Jheng-Yan Wu, Chia-Hung Yu, Ying-Jen Chang, Kuo-Chuan Hung

**Affiliations:** ^1^ Department of Anesthesiology, Chi Mei Medical Center, Liouying, Tainan, Taiwan; ^2^ Department of Anesthesiology, E-Da Hospital, I-Shou University, Kaohsiung, Taiwan; ^3^ Department of Nutrition, Chi Mei Medical Center, Tainan, Taiwan; ^4^ Department of Anesthesiology, Chi Mei Medical Center, Tainan, Taiwan

**Keywords:** triglyceride-glucose index, TyG, cancers, sensitivity, specificity, prediction

## Abstract

**Objective:**

This meta-analysis aimed to evaluate the diagnostic performance of the triglyceride-glucose (TyG) index in predicting cancer occurrence.

**Method:**

A comprehensive literature search was conducted in Embase, Medline, Cochrane Library, and Google Scholar from inception to July 2024. Observational studies reporting the diagnostic efficacy of the TyG index in predicting cancer occurrence using ROC curve analysis were included. Pooled sensitivity, specificity, and area under the summary receiver operating characteristic (SROC) curve were calculated using a bivariate random-effects model.

**Results:**

Eleven studies with 46,658 participants were included. Patients with cancer had a significantly higher TyG index than those without cancer (mean difference: 0.34, 95% CI: 0.23-0.45). The pooled sensitivity and specificity of the TyG index for predicting cancer occurrence were 0.68 (95% CI: 0.62-0.74) and 0.65 (95% CI: 0.54-0.74), respectively. The area under the SROC curve was 0.72 (95% CI: 0.68-0.75), indicating good discriminatory ability. Subgroup analysis of female participants yielded similar results, with an AUC of 0.73 (95% CI: 0.69-0.77).

**Conclusion:**

The TyG index demonstrates good discriminatory ability and may have potential as an adjunct screening tool to help identify individuals at a higher risk of developing cancer. However, this should be interpreted alongside other established risk factors, as many confounding factors (including cancer type, genetic predisposition, and other malignancy risk factors) must be considered. Further research is needed to establish optimal cut-off values, which likely vary across different cancer types, and to investigate their diagnostic accuracy in diverse populations.

**Systematic review registration:**

https://www.crd.york.ac.uk/prospero/, identifier CRD42024573712.

## 1 Introduction

Cancer remains a major global health issue, with 19.3 million new cases and nearly 10 million deaths worldwide in 2020 (1). The global cancer burden is anticipated to increase by 47% to 28.4 million cases by 2040, with transitioning countries experiencing a more pronounced increase due to demographic shifts and increasing risk factors (1), emphasizing the need for sustainable cancer prevention and care infrastructure in these regions. Early detection and prevention strategies are crucial for improving outcomes and reducing global cancer burden. Metabolic syndrome, a cluster of metabolic disorders, is associated with numerous types of cancer (2–6). Insulin resistance, a pivotal factor in the development of metabolic syndrome, is believed to play a significant role in the development of numerous types of cancer through multiple complex pathways, including hyperinsulinemia, increased bioavailability of insulin-like growth factor-1 (IGF-1), and chronic inflammation (7–10). Additionally, changes in gastrointestinal hormones, such as ghrelin, GLP-1, and PYY, have been observed in metabolic syndrome and obesity, with emerging evidence suggesting that ghrelin may be specifically associated with cancer proliferation and metastasis (11). In recent years, there has been growing interest in exploring the relationship between metabolic disorders and cancer risk, with particular attention given to insulin resistance and its associated biomarkers (7, 12, 13).

The triglyceride-glucose (TyG) index is a surrogate marker for insulin resistance and a valuable tool for determining metabolic syndrome (14, 15). Originally proposed to identify individuals at risk for cardiovascular diseases and type 2 diabetes (16–19), recent studies have suggested that the TyG index may also have the potential to predict cancer occurrence (20–22). Although a previous meta-analysis of six observational studies indicated that a higher TyG index was associated with a slightly increased cancer risk compared to a lower TyG index (total effect size = 1.14) (23), the diagnostic performance of the TyG index in predicting cancer was not assessed. Analyzing the diagnostic efficacy of the TyG index can aid healthcare professionals in comprehending its advantages and drawbacks, which could lead to its implementation as an initiative to detect individuals with a heightened likelihood of developing cancer, thus enabling them to adjust their lifestyles and take preventive measures at an earlier stage. Additionally, the widespread availability and ease of calculation of the TyG index make it an accessible tool for large-scale screening programs, which could lead to better population health management and reduced healthcare costs.

To address this knowledge gap, we conducted a comprehensive meta-analysis to synthesize the available evidence on the diagnostic performance of the TyG index in cancer prediction. By consolidating and analyzing the existing literature, this study sought to provide valuable insights into the utility of the TyG index as a potential screening tool for cancer risk assessment and to guide future research directions in this promising field.

## 2 Method

### 2.1 Search strategy

This study was officially registered with PROSPERO under the identifier CRD42024573712. We followed the PRISMA guidelines for methodology and reporting, which ensured a thorough evaluation of the quality of the systematic reviews included in this meta-analysis.

We conducted a literature search to identify studies exploring the relationship between the TyG index and cancer occurrence. The search included Embase, Medline, and Cochrane Library from their inception to July 2024, using both Medical Subject Headings (MeSH) terms and free-text keywords related to the TyG index and cancer. The search terms, which included “triglyceride glucose index,” “TyG index,” “triglyceride-glucose index,” “triglyceride to glucose index,” as well as cancer-related terms such as “neoplasm*,” “cancer*,” “malignan*,” “tumor*,” and “carcinoma*,” were combined using Boolean operators “AND” and “OR.” The complete Medline search strategy is detailed in **Table 1**, with adaptations to other databases. To ensure thorough coverage, we manually searched the reference lists of included studies and relevant review articles. In addition, we also explored gray literature using Google Scholar, which provides access to a wide range of publications, including conference papers, theses, and institutional repositories. We did not include congress abstracts in our analysis because of the limited information they provided for quality assessment.

**Table 1:** Search strategy for Medline.

#	Search syntax
1	(“triglyceride glucose index” OR “TyG index” OR “triglyceride glucose indices” OR “triglyceride to glucose index” OR “triglyceride-to-glucose index” OR “Triglyceride/glucose index”).mp
2	(“cancer” OR “tumor” OR “neoplasms” OR “malignant neoplasm” OR “carcinoma”).mp
3	exp “Neoplasms”/OR exp “Carcinoma”/
4	(1) AND (2 OR 3)

Two independent reviewers (I.-W.C. and Y.-J.C.) screened the titles and abstracts of all identified articles, followed by the retrieval and assessment of the full texts of potentially eligible studies against the inclusion and exclusion criteria. Any disagreements were resolved through discussion with a third reviewer (K.-C.H.).

### 2.2 Inclusion criteria

Studies were eligible for inclusion if they were (1) original research articles published in peer-reviewed journals without language limitations; (2) observational studies (i.e., cohort, case-control, or cross-sectional) that reported the diagnostic performance of the TyG index in the prediction of cancer occurrence using ROC curve analysis; and (3) provided sufficient data (sensitivity, specificity, and patient counts with or without cancer) to calculate relevant information (true positive, false negative, false positive, true negative).

Excluded studies were reviews, meta-analyses, case reports, editorials, studies focusing solely on cancer prognosis or mortality without data on cancer occurrence, studies on the development of cancer metastasis or non-invasive cancer (e.g., colon adenoma), and studies with overlapping patient populations, in which case the study with the largest sample size or the most comprehensive data was included.

### 2.3 Data extraction

Two independent reviewers (I.-W.C. and Y.-J.C.) used a standardized form to extract data from the included studies, and any discrepancies were resolved through discussion. Data extraction included study characteristics (first author, publication year, country, study design, and sample size), participant characteristics (age, sex, and body mass index), and TyG index (data (cut-off values used). Other details on sensitivity, specificity, and patient counts with or without cancer were also recorded. The authors of the included studies were contacted for any missing data or clarification. All extracted data were entered into a spreadsheet and checked for accuracy by a third reviewer (K.-C.H.).

### 2.4 Outcomes and measurement

The TyG index was calculated as ln [fasting triglycerides (mg/dL) × fasting glucose (mg/dL)/2]. This dimensionless index typically ranges from approximately 4 to 10 in clinical settings, with higher values indicating greater insulin resistance. The primary outcome of interest was the diagnostic performance of the TyG index in predicting cancer occurrence. To measure the diagnostic performance of the TyG index, we used the summary receiver operating characteristic (SROC) curve approach.

### 2.5 Risk of bias assessment

The risk of bias in the included studies was assessed using the QUADAS-2 tool (24), with two independent reviewers (I.-W.C. and Y.-J.C.) evaluating each study across four key domains: patient selection, index test (TyG index), reference standard (cancer diagnosis), and flow and timing. Each domain was assessed for risk of bias, and the first three domains were also assessed for concerns regarding applicability to the research question. Signaling questions guided the assessment, and the risk of bias and applicability concerns were judged as “low,” “high,” or “unclear” for each domain.

### 2.6 Statistical analysis

Statistical analysis was performed using the “mada” package in R (version 4.0.5) and the Cochrane Review Manager (RevMan 5.3; Copenhagen: The Nordic Cochrane Center, The Cochrane Collaboration, 2014). A random-effects model was used for the meta-analysis. Diagnostic accuracy measures, including sensitivity, specificity, and their corresponding 95% confidence intervals (CIs), were calculated for each included study. The pooled estimates of these measures were obtained using a bivariate random-effects model. Heterogeneity among the studies was assessed using the I² statistic. An I² value > 75% was considered indicative of significant heterogeneity. To assess whether the study design influenced the observed TyG index differences between cancer and non-cancer patients, we conducted a subgroup analysis categorizing studies by their methodological approach (cross-sectional, case-control, and cohort studies). An SROC curve was constructed to evaluate the overall diagnostic accuracy of the TyG index in predicting cancer occurrence. The SROC curve is commonly used in meta-analyses of diagnostic test accuracy. It plots the true positive rate (sensitivity) against the false positive rate (1-specificity) from multiple studies, providing a global summary of test performance. The area under the SROC curve (AUC) was calculated with its corresponding 95% CI. An AUC value of 0.5 indicates no discriminatory ability, while a value of 1.0 represents perfect discrimination. An AUC value ≥ 0.7 will be considered indicative of a useful diagnostic method (25), as it suggests that the TyG index has good discriminatory ability in predicting cancer occurrence. Publication bias was assessed using Deek’s funnel plot asymmetry test. A P-value < 0.1 for Deeks’ test was considered indicative of significant publication bias. If feasible, subgroup analyses were performed based on sex to explore potential sources of heterogeneity and to assess the diagnostic performance of the TyG index in predicting cancer occurrence separately for males and females. The same meta-analytic methods described above were applied to the subgroup data. All statistical tests will be two-tailed, and a P-value < 0.05 will be considered statistically significant.

## 3 Results

### 3.1 Characteristics of studies

**Figure 1:**
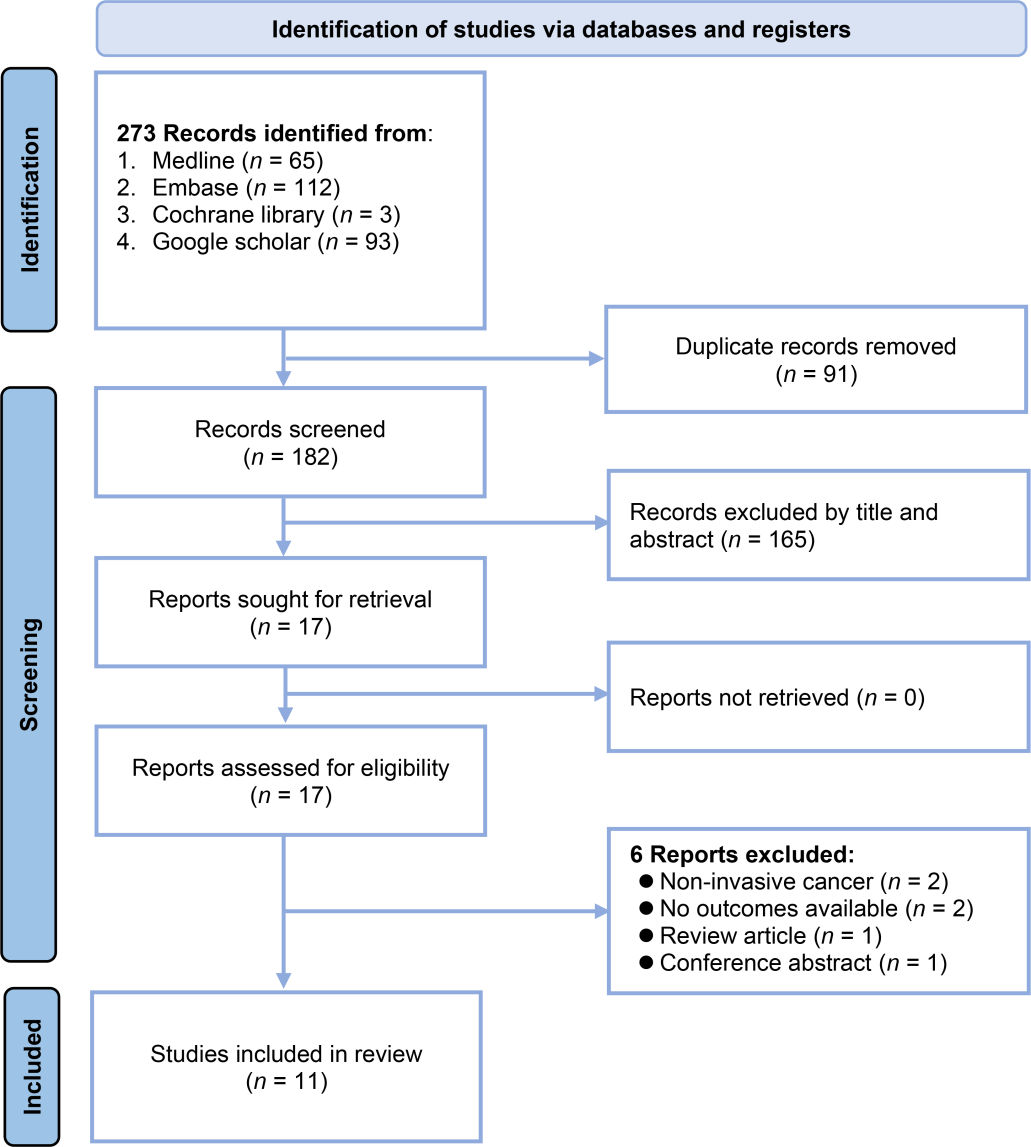
Flowchart of study selection.

The study selection process is depicted in **Figure 1**. A total of 273 records were initially identified by searching the following databases: Medline (n = 65), Embase (n = 112), Cochrane library (n = 3), and Google Scholar (n = 93). After removing 91 duplicate records, 182 unique records were screened based on their titles and abstracts. This screening excluded 165 records that did not meet the eligibility criteria. The full texts of the remaining 17 reports were sought for a detailed evaluation. Of these, six reports were excluded for the following reasons: two reports had non-invasive cancer as the outcome, two had no outcomes available, one was a review article, and one was a conference abstract. Consequently, 11 studies with 46,658 patients were included in the systematic review (20–22, 26–30, 32–34).

The main characteristics of the 11 studies included in this systematic review are summarized in **Table 2**. The publication period ranged from 2020 to 2024. The studies were conducted in various countries, including six in China (21, 28, 30, 32–34), three in Turkey (20, 26, 27), one in Japan (22), and one in India (29). The sample sizes of the included studies ranged from 200 to 27,921 participants. The mean or median age of the study population varied from 41 to 71 years. Six studies included both male and female participants, whereas five studies included only females (21, 27, 29, 30, 33). The percentage of males in the studies that included both sexes ranged from 23% to 100%. The cut-off value for the TyG index to diagnose cancer ranged from 4.49 to 9.091. The AUC was reported in all studies as a measure of the diagnostic accuracy of the TyG index in predicting cancer occurrence. The AUC values ranged from 0.593 to 0.835, with six studies reporting an AUC ≥ 0.7 (21, 26, 28–30, 32), indicating a fair to good discriminatory ability. The included studies investigated the diagnostic efficacy of the TyG index in predicting various types of cancer, including colorectal cancer (two studies), prostate cancer (two studies), thyroid cancer (one study), gynecologic/breast cancer (five studies), and lung cancer (one study).

**Table 2:** Characteristics of the included studies.

Studies	Age (years)	Male (%)	n	BMI	Cut-off	AUC	Type of cancer	Study design	Country
Aksoy, 2024 (26)	62/60	52/55	256	28/27	4.49	0.782	Colorectal Ca	Case-control	Turkey
Alkurt 2022a (20)	57/46	0	510	na	8.628	0.606	Breast Ca	Cross-sectional study	Turkey
Alkurt 2022b (27)	50/52	23/31	382	na	6.252	0.608	Thyroid Ca	Cross-sectional study	Turkey
Li 2023 (28)	71	100	767	24	8.497	0.758	Prostate Ca	Case-control	China
Okamura 2020 (22)	46	59	27921	23	8.272	0.687	Colorectal Ca	Longitudinal cohort study	Japan
Rajakumar 2024† (29)	55/29	0	200	25/25	8.95	0.835	Breast ca	Cross-sectional study	India
Shi 2024 (30)	54	0	674	27-31	8.02	0.78	Endometrial Ca	Retrospective cohort study	China
Shi 2022 (31)	41-55	0	11466	na	8.7	0.726	Gynecologic/breast Ca	Cross-sectional study	China
Yan 2021 (32)	62/60	43	1578	24/23	8.18	0.713	Lung Ca	Case-control study	China
Zhang 2024 (33)	51/40	0	2588	23/22	8.12	0.608	Breast Ca	Cross-sectional study	China
Zhou 2023 (34)	71/65	100	316	24/24	9.091	0.593	Prostate Ca	Retrospective study	China

†prospective; BMI, body mass index; AUC, area under curve; Ca, cancer; na, not available.

### 3.2 Risk of bias

**Figure 2:**
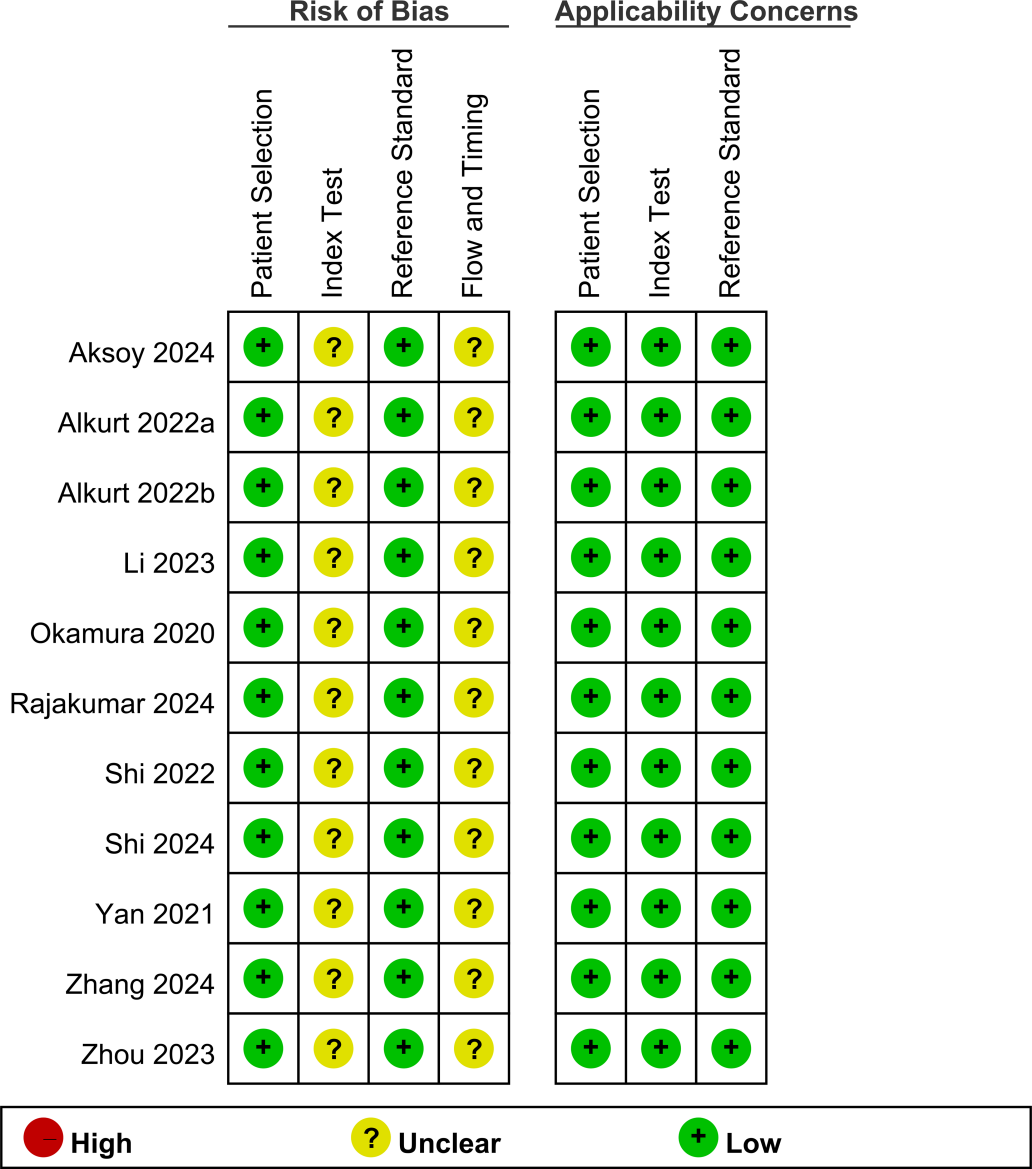
Quality of studies.

The results of the risk of bias assessment are shown in **Figure 2**. All studies had a low risk of bias in the patient selection domain. Regarding the index test, all studies had an unclear risk of bias owing to the absence of a predefined cutoff value for the TyG index in cancer diagnosis. For the reference standard domain, all studies were judged to have a low risk of bias. In the flow and timing domains, all studies had an unclear risk. Concerning applicability, all studies were assessed as having a low risk of bias in the patient selection, index test domains, and reference standard domain.

### 3.3 Outcomes

#### 3.3.1 TyG index in patients with or without cancer

**Figure 3:**
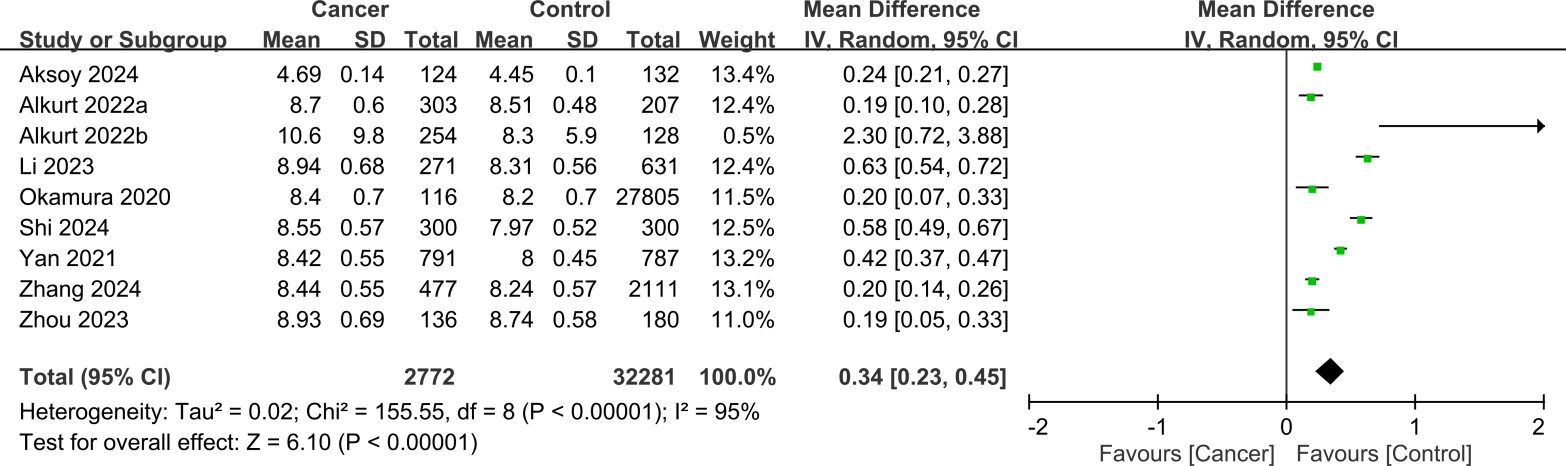
Forest plot showing the mean difference in triglyceride-glucose (TyG) index between patients with and without cancer. IV, invariance; CI, confidence interval.

The mean difference in the TyG index between the cancer and control groups was calculated for each study and overall (**Figure 3**) (20, 22, 26–28, 30, 32–34). The mean TyG index values ranged from 4.69 to 10.6 in the cancer groups and from 4.45 to 8.74 in the control groups across the individual studies. The mean difference in the TyG index between the cancer and control groups varied among the included studies, ranging from 0.19 to 2.3. The pooled mean difference in TyG index was 0.34 (95% CI: 0.23-0.45), indicating that participants with cancer had, on average, a 0.34 higher TyG index compared to those without cancer.

**Figure 4:**
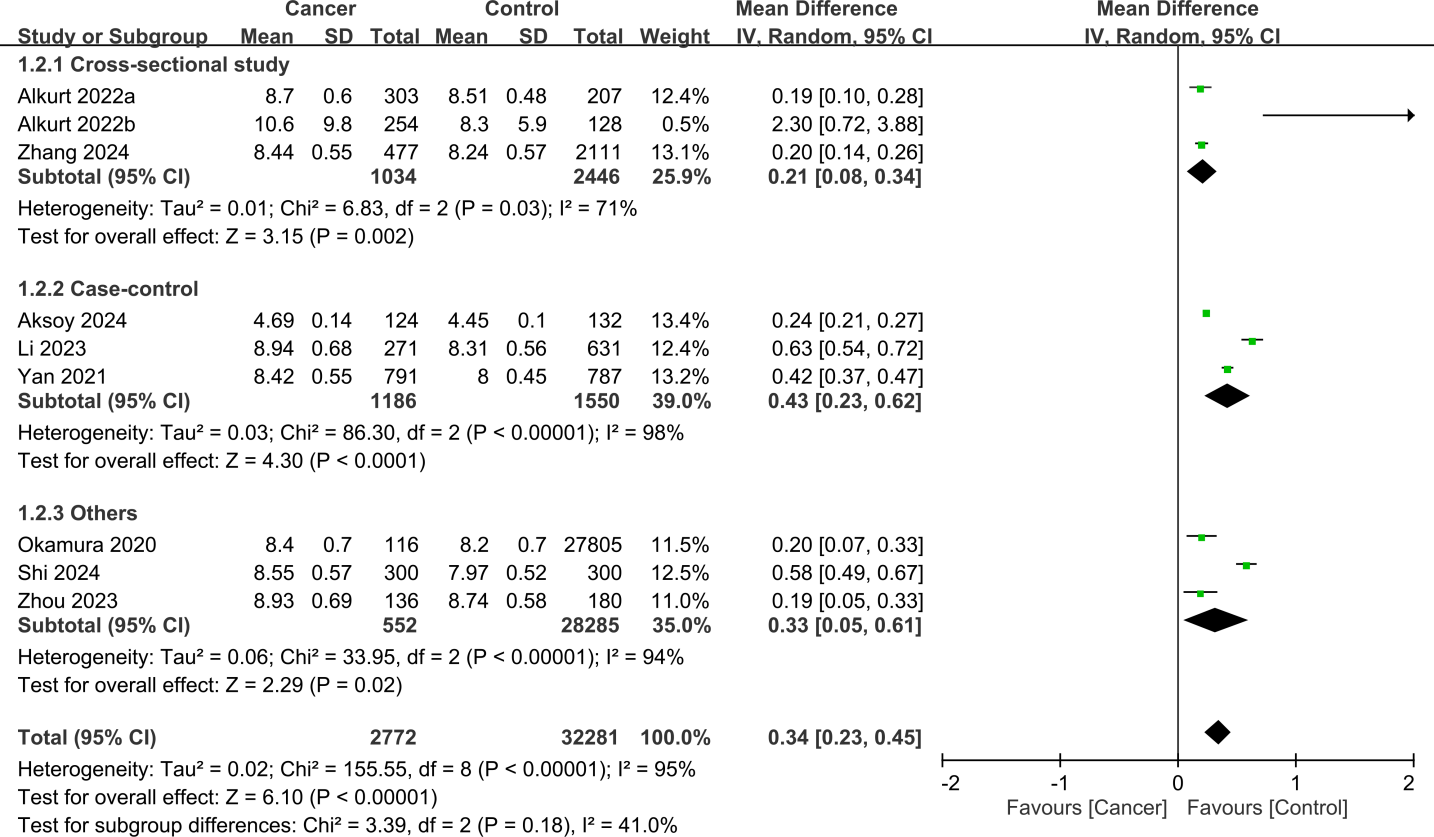
Forest plot showing the mean difference in triglyceride-glucose (TyG) index between the cancer and control groups stratified by study design. No significant subgroup difference was detected between the study designs (p=0.18). IV, inverse variance; CI, confidence interval.

**Figure 4** shows the mean differences in the TyG index between cancer and non-cancer participants stratified by study design. Cross-sectional studies showed a pooled mean difference of 0.21 (95% CI: 0.08-0.34, I²=71%), indicating that cancer patients had on average a 0.21 higher TyG index compared to those without cancer. Case-control studies demonstrated a larger mean difference of 0.43 (95% CI: 0.23-0.62, I²=98%), while cohort studies showed an intermediate difference of 0.33 (95% CI: 0.05-0.61, I²=94%). No subgroup differences were noted (p=0.18).

#### 3.3.2 Sensitivity and specificity analysis

**Figure 5:**
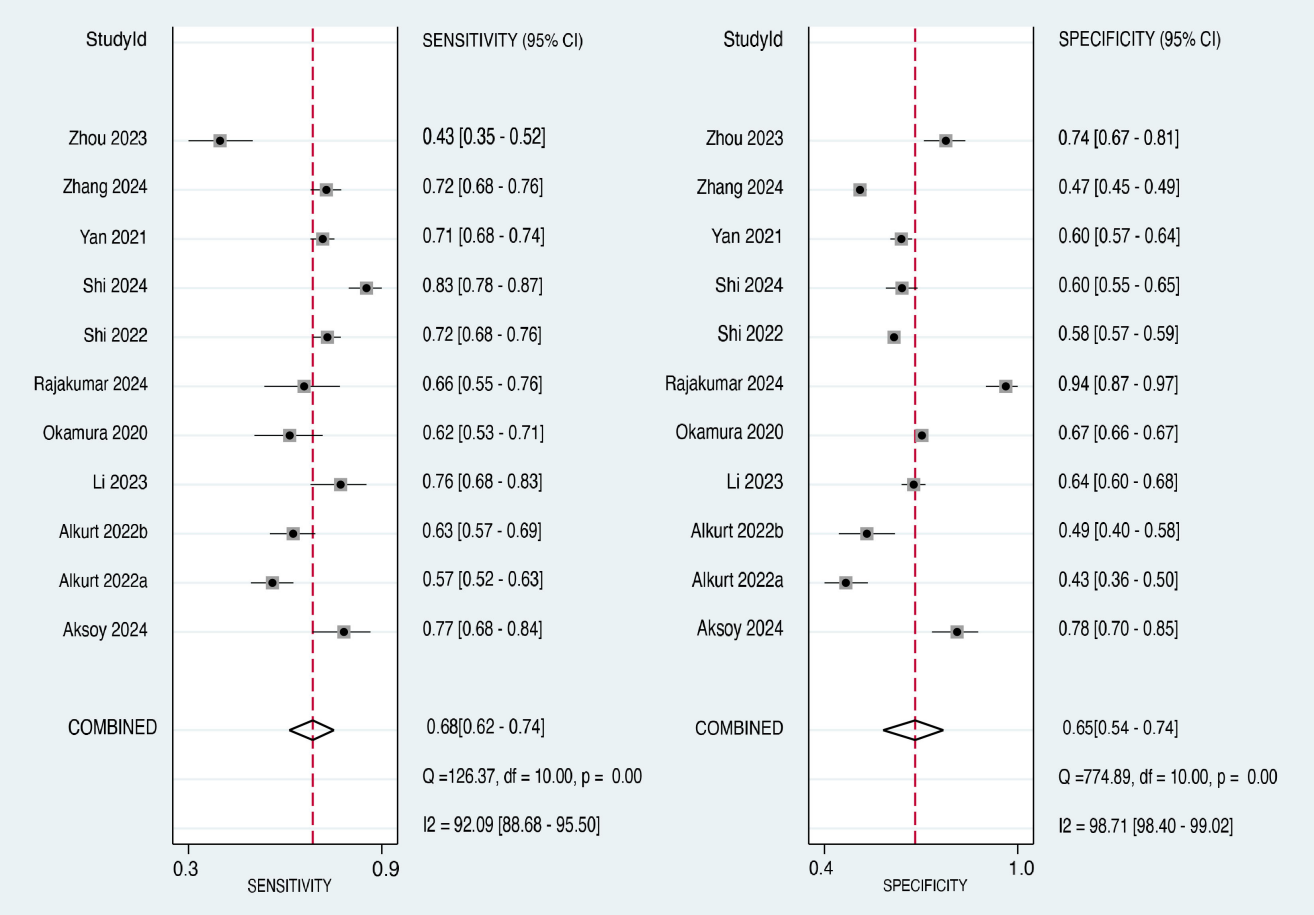
Forest plot showing pooled sensitivity and specificity in 11 studies.

The forest plot of the sensitivity estimates for each included study and the pooled sensitivity are presented in the left panel of **Figure 5** (20–22, 26–30, 32–34). The sensitivity values ranged from 0.43 to 0.83. The pooled sensitivity across all 11 studies was 0.68 (95% CI: 0.62-0.74), indicating that the TyG index correctly identified 68% of individuals who developed cancer. The I² value was 92.09%, suggesting a high degree of heterogeneity in the sensitivity estimates across studies.

The forest plot of the specificity estimates for each included study and the pooled specificity are presented in the right panel of **Figure 5** (20–22, 26–30, 32–34). The specificity values ranged from 0.43 to 0.94. The pooled specificity across all 11 studies was 0.65 (95% CI: 0.54-0.74), indicating that the TyG index correctly identified 65% of individuals who did not develop cancer. Similar to the sensitivity analysis, the I² value was 98.71%, suggesting a high degree of heterogeneity in the specificity estimates across studies.

#### 3.3.3 ROC curve analysis

**Figure 6:**
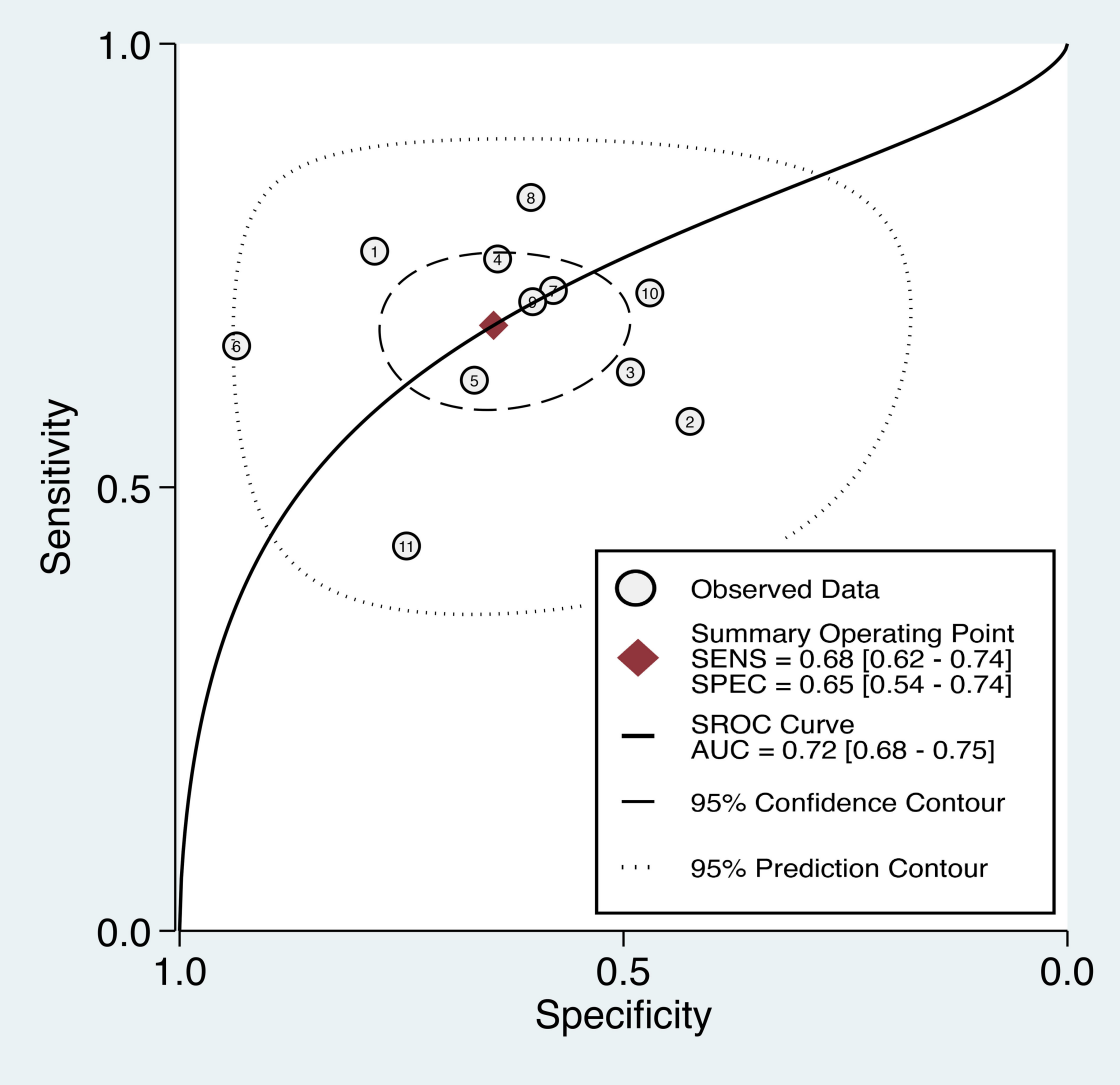
The summary receiver operating characteristic (sROC) curve analysis showed the di-agnostic performance of the triglyceride-glucose (TyG) index in predicting cancer occurrence. AUC, area under the curve; SENS, sensitivity; SPEC, specificity.

The overall diagnostic performance of the TyG index in predicting cancer occurrence was assessed using the SROC curve, which illustrates the relationship between sensitivity and false-positive rate (1-specificity) for each included study. The AUC was 0.72 (95% CI: 0.68-0.75) (**Figure 6**), indicating that the TyG index has a good discriminatory ability in distinguishing between individuals who developed cancer and those who did not. Therefore, there is a 72% probability that a randomly chosen individual with cancer will have a higher TyG index value than that of a randomly chosen individual without cancer.

#### 3.3.4 Publication bias

**Figure 7:**
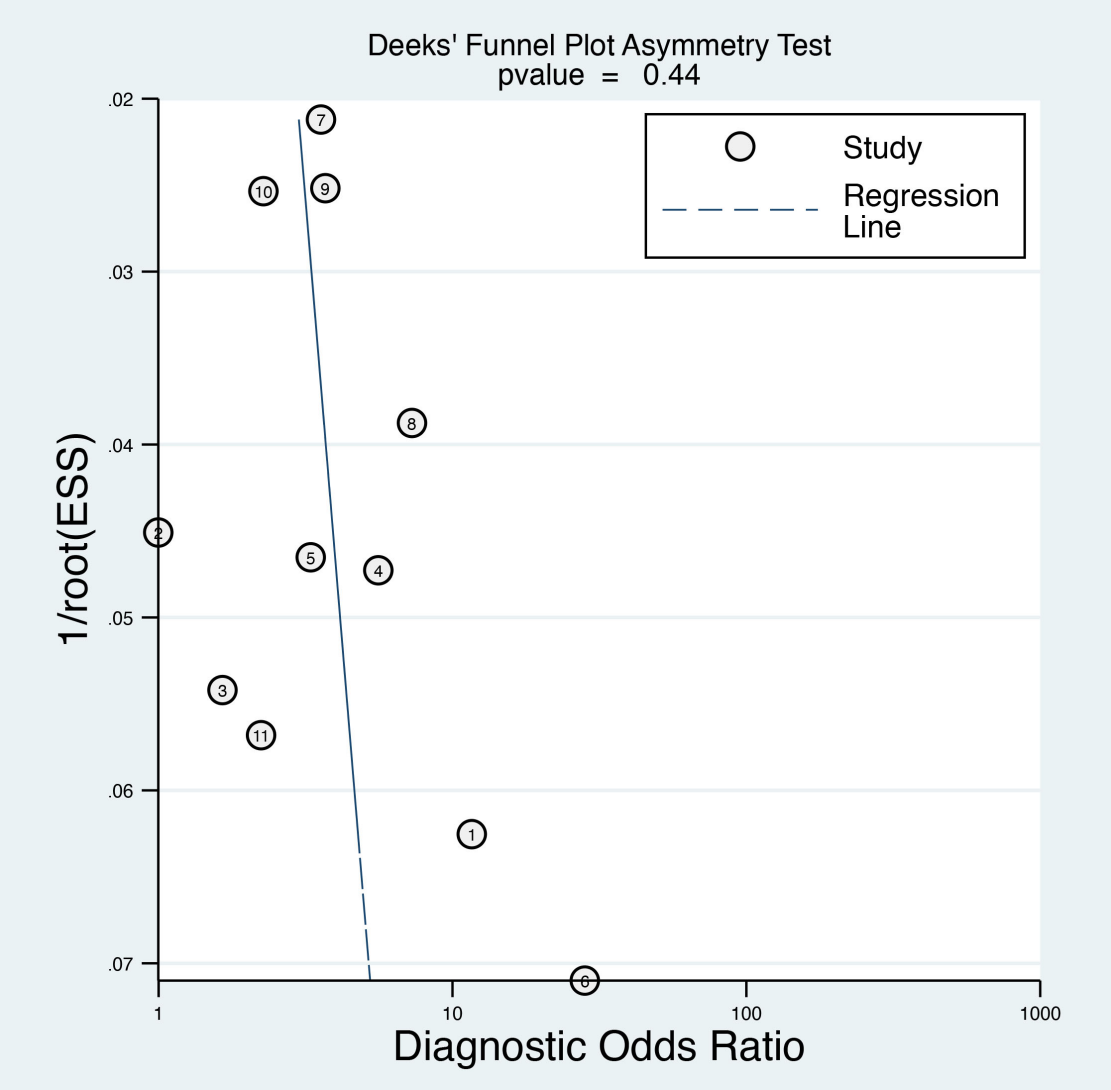
Deek’s funnel plot asymmetry test was performed to evaluate publication bias among the included studies, which indicated a low risk of bias.

The Deeks’ funnel plot asymmetry test yielded a p-value of 0.44 (**Figure 7**), which is above the conventional threshold of 0.10 used to indicate significant publication bias. This finding suggests that there is no strong evidence of publication bias influencing the results of this meta-analysis.

#### 3.3.5 Subgroup analysis on female patients

**Figure 8:**
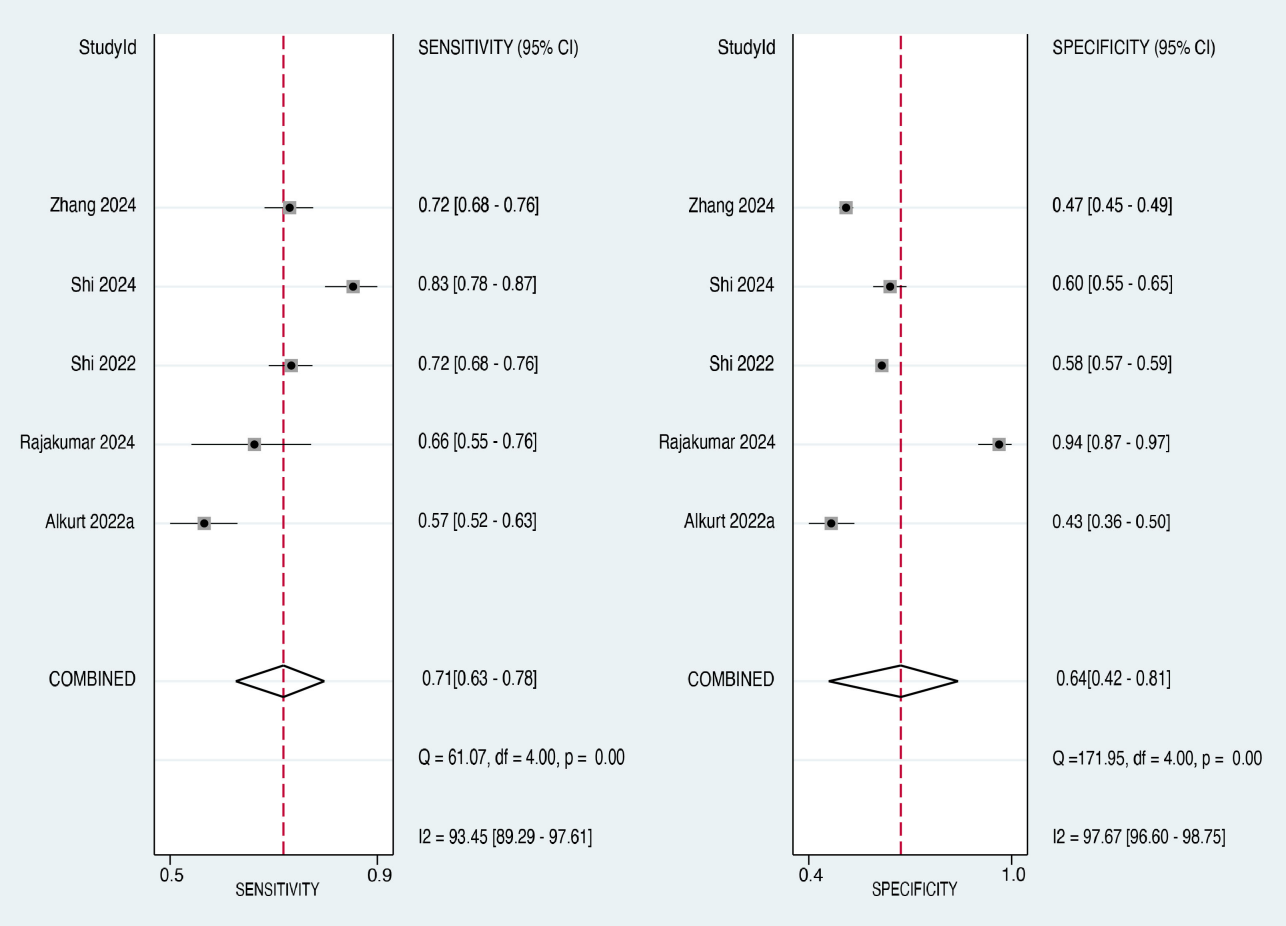
Subgroup analysis showing the pooled sensitivity and specificity in five studies.

Subgroup analysis was performed to evaluate the diagnostic accuracy of the TyG index in predicting cancer occurrence, specifically in female patients. Five studies (21, 27, 29, 30, 33) provided data on the sensitivity and specificity of the TyG index in female participants. The forest plot of the sensitivity estimates for the female subgroup is presented in the left panel of **Figure 8**. The sensitivity values ranged from 0.57 to 0.83, with a pooled sensitivity of 0.71 (95% CI: 0.63-0.78). The I² value was 93.45%, suggesting a high degree of heterogeneity in the sensitivity estimates across studies.

**Figure 9:**
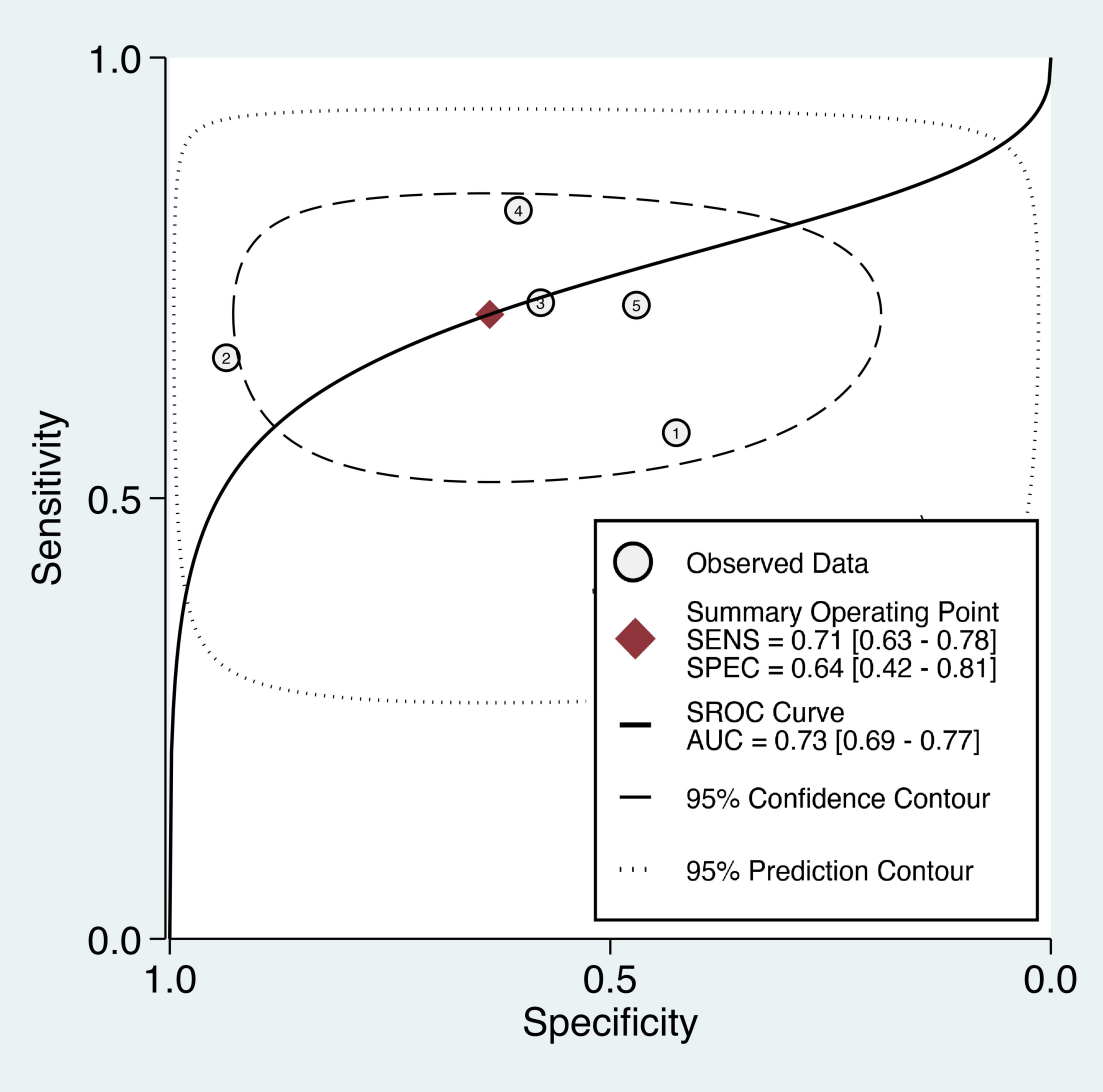
Subgroup analysis of female patients showing the diagnostic performance of the triglyceride-glucose (TyG) index in predicting cancer occurrence. AUC, area under the curve; SENS, sensitivity; SPEC, specificity.

The forest plot of specificity estimates for the female subgroup is presented in the right panel of **Figure 8**. The specificity values ranged from 0.43 to 0.94, with a pooled specificity of 0.64 (95% CI: 0.42-0.81). Heterogeneity among the studies in the female subgroup was high, with an I² value of 97.67%, confirming significant heterogeneity in the specificity estimates. The overall diagnostic accuracy of the TyG index for predicting cancer occurrence in the subgroup of female patients was evaluated using the SROC curve (**Figure 9**). The AUC for the female subgroup was 0.73 (95% CI: 0.69-0.77), indicating that the TyG index has a good discriminatory ability in distinguishing between female individuals who developed cancer and those who did not.

## 4 Discussion

The meta-analysis included 11 observational studies that reported the diagnostic efficacy of the TyG index in predicting cancer occurrence using ROC curve analysis. The pooled mean difference in TyG index between cancer and control groups was 0.34 (95% CI: 0.23-0.45), indicating that participants with cancer had, on average, a 0.34 higher TyG index compared to those without cancer. The pooled sensitivity and specificity of the TyG index in predicting cancer occurrence were 0.68 (95% CI: 0.62-0.74) and 0.65 (95% CI: 0.54-0.74), respectively. The area under the SROC curve was 0.72 (95% CI: 0.68-0.75), indicating that the TyG index has an acceptable discriminatory ability in distinguishing between individuals who developed cancer and those who did not. A subgroup analysis of female patients yielded similar results, with an AUC of 0.73 (95% CI: 0.69-0.77), suggesting that the TyG index may be a useful tool for predicting cancer occurrence in both the general population and specifically in women.

There are several methods to measure insulin resistance, broadly classified into direct and indirect measurements. The gold standard for direct measurement is the hyperinsulinemic-euglycemic clamp technique (35), although it is invasive, time-consuming, and expensive, rendering it impractical for large-scale epidemiological studies or routine clinical use. Indirect measurements of insulin resistance can be divided into insulin- and non-insulin-based indices. Insulin-based indices, such as the homeostatic model assessment for insulin resistance (HOMA-IR) (36), require fasting insulin and glucose measurements. Although simpler than the clamp technique, these indices still involve insulin level measurements, which may not be available in all settings. Non-insulin-based indices provide a more practical approach for assessing insulin resistance in large populations. These included the TyG index, combination of TyG and body mass index (TyG-BMI), triglyceride to high-density lipoprotein cholesterol ratio (TG/HDL-C ratio), and metabolic score for insulin resistance (METS-IR) (37–43). These indices are derived from commonly measured clinical variables, such as fasting glucose, triglycerides, HDL-cholesterol, and BMI, making them more accessible and cost-effective.

Several studies have investigated the association between insulin resistance and cancer occurrence, providing evidence of a potential link between these two conditions. A meta-analysis of twenty-two studies involving 33,405 participants found that HOMA-IR levels were significantly higher in breast cancer patients than in individuals without breast cancer (44). Similarly, two previous meta-analyses revealed significant associations between HOMA-IR levels and endometrial cancer or colorectal adenomas (45, 46). It’s important to note that obesity and metabolic syndrome create a chronic inflammatory state that may contribute to an environment promoting malignancy development (47). For context, TyG index values ≥8.5-8.8 are typically considered indicative of insulin resistance in adults (48), though optimal cut-offs for cancer prediction may differ. Recently, a meta-analysis of six studies indicated that a higher TyG index may be associated with an increased risk of cancer (23). Although the meta-analysis by Wang et al. (23) established a correlation between an elevated TyG index and increased cancer risk, our research goes a step further by focusing on the diagnostic performance of the TyG index using ROC curve analysis. Our study represents a significant advancement in this field, as it is the first to systematically evaluate the diagnostic efficacy of the TyG index for cancer prediction. This approach allows us to determine not only whether the TyG index is associated with cancer risk but also how well it can discriminate between individuals who will develop cancer and those who will not.

It is also important to acknowledge that while our meta-analysis demonstrated an AUC of 0.72 for the TyG index in predicting cancer occurrence, recent interpretative guidelines suggest that diagnostic tests with AUC values below 0.80 should be considered with caution in clinical practice (49). In addition, the lower bound of our CI (0.68) falls below the conventional threshold of 0.70 for a useful diagnostic test. Therefore, it is important to emphasize that, while our findings suggest the potential utility of the TyG index in cancer risk assessment, it should be considered as an adjunctive tool rather than a standalone predictor. The relationship between insulin resistance and cancer development is complex and likely modulated by numerous confounding factors that must be considered when evaluating cancer risk. These include cancer type-specific risk factors, genetic predisposition, family history, environmental exposure, and lifestyle factors. The lack of established threshold values across different populations and cancer types further underscores the need for careful interpretation of TyG index values in clinical settings.

Our findings indicate that the TyG index could potentially be used as an adjunct screening tool to help identify individuals at a higher risk of developing cancer. However, its moderate sensitivity (0.68, 95% CI: 0.62-0.74) and specificity (0.65, 95% CI: 0.54-0.74) indicate that while valuable, the TyG index alone may not be sufficient for accurate cancer prediction. Integrating the TyG index with other established risk factors and biomarkers may yield more robust predictive models to enhance the efficacy of cancer prediction. For example, chronic inflammation plays a crucial role in cancer development (50, 51). A previous study reported that combining the TyG index with inflammatory markers such as the platelet-to-lymphocyte ratio (PLR) could enhance the predictive power of cancer risk assessment models (29). In addition, combining the TyG index with cancer-specific biomarkers may improve early detection rates and reduce false positives. Another study reported that the TyG index combined with the initial prostate-specific antigen (PSA) level and age can improve diagnostic efficacy (34). We suggest that the TyG index should not be used as a standalone diagnostic tool and should be interpreted alongside other clinical factors and diagnostic tests.

Our subgroup analysis by study design revealed variations in the magnitude of the TyG index differences between cancer and non-cancer patients across different methodological approaches. The finding that these variations did not reach statistical significance (p=0.18) suggests that the association between elevated TyG index and cancer is relatively consistent, regardless of the study design. The absence of significant subgroup differences strengthens the robustness of our findings, indicating that the relationship between the TyG index and cancer is not substantially influenced by the methodological approach. This consistency across various study designs provides more reliable evidence for the association between insulin resistance (as measured by the TyG index) and cancer.

The findings of this meta-analysis may have implications for public health policies. Given the potential of the TyG index to identify individuals at a higher risk of developing cancer, policymakers could consider incorporating the TyG index into population-level cancer screening programs. This could help to target screening efforts towards individuals who are most likely to benefit, potentially improving the cost-effectiveness of screening programs. However, before implementing such policies, further research is needed to establish the optimal cutoff values for the TyG index in different populations, and to evaluate the feasibility and acceptability of using the TyG index in population-level screening programs. Policymakers should also consider the potential harms of using the TyG index for cancer screening, such as the risk of false-positive results, leading to unnecessary further testing or anxiety in patients.

This meta-analysis has several limitations that warrant consideration. First, the significant heterogeneity observed among the included studies, likely stemming from variations in study populations, cancer types, and TyG index cutoff values, may affect the generalizability of our findings. In addition, the geographical bias towards Asian countries, particularly China and Turkey, limits the applicability of our results to more diverse global populations. Second, the lack of standardized TyG index cut-off values (ranging from 4.49 to 9.091) posed challenges in establishing a universally applicable threshold for cancer risk prediction. Third, the paucity of data on male populations prevented comprehensive sex-specific analysis, limiting our understanding of the potential sex differences in the TyG index’s predictive ability. Fourth, potential confounding factors such as diet, physical activity, and other metabolic parameters may not have been adequately controlled for in all studies. Additionally, the potential influence of concurrent metabolic conditions, such as diabetes or metabolic syndrome, on the relationship between the TyG index and cancer risk was not fully explored in this meta-analysis. Fifth, most studies employed a cross-sectional design, which may have affected the assessment of cancer occurrence and limited the TyG index’s long-term predictive value. Finally, the lack of data on cancer stage and aggressiveness at diagnosis limits our ability to assess the clinical utility of the TyG index as a cancer prediction tool.

## 5 Conclusion

This meta-analysis revealed that the TyG index has an acceptable discriminatory ability that could assist in predicting cancer occurrence in both the general population and female patients when used as part of a comprehensive risk assessment approach. Despite showing promise, the TyG index should be considered an adjunctive tool rather than a definitive predictor of cancer risk. The limitations of heterogeneity, absence of predefined cutoff values that likely vary across cancer types, and potential influence of numerous confounding factors (including cancer type, genetic predisposition, and other established risk factors) necessitate cautious interpretation. Future research should focus on establishing optimal cutoff values for specific cancer types, considering factors such as age, sex, and ethnicity, and exploring the potential of combining the TyG index with other biomarkers or risk factors to enhance its predictive value in integrated risk assessment models.

## Data Availability

The original contributions presented in the study are included in the article/supplementary material. Further inquiries can be directed to the corresponding author.
